# Ocular drug delivery systems: glaucoma patient perceptions from a German university hospital eye clinic

**DOI:** 10.1007/s00417-023-06248-1

**Published:** 2023-09-30

**Authors:** Constance Weber, Philipp Quintin, Frank G. Holz, Antonio Fea, Karl Mercieca

**Affiliations:** 1 Department of Ophthalmology, University of Bonn, Ernst-Abbe-Straße 2, 53117 Bonn, Germany; 2 Department of Ophthalmology, University of Turin, Turin, Italy

**Keywords:** Ocular drug delivery devices, Stents, Glaucoma medication, Patient adherence, Eyedrops

## Abstract

**Purpose:**

This study aimed to report on glaucoma patients’ beliefs and illness perceptions and to investigate their opinion on ocular drug delivery devices (ODD).

**Methods:**

We performed a cross-sectional study in a large tertiary-referral outpatient glaucoma clinic, with 102 patients. Validated anonymized questionnaires were used. We investigated the awareness and acceptance regarding ODD (contact lenses (CLs), punctal plugs (PPs), subconjunctival implants, anterior chamber (AC) injections, and drug-emitting stents) and looked at factors that could influence a patient’s decision for having an ODD.

**Results:**

Sixty-three patients (61.8%) confirmed they would rather have ODD than keep their eye-drops (38.2%). The most important factors influencing their decision were effectiveness and long-lasting effect. A large proportion of patients reported a preference for CLs (48.0%), PPs (52.9%), or drug-emitting stents (44.1%). When comparing patients who preferred ODD (group-1) versus eye-drops (group-2), significantly more patients in group-1 were worried (*p* < 0.001) or felt disrupted (*p* < 0.001) by their use of eye-drops. A significantly greater share of patients in group-1 showed acceptance towards CLs (60.3% vs. 38.5%; *p* = 0.032), AC injections (38.1% vs. 12.8%, *p* = 0.006), or drug-emitting stents (54% vs. 28.2%, *p* = 0.023), whilst there were no significant differences regarding the acceptance of PPs (*p* = 0.363) or subconjunctival implants (*p* = 0.058).

**Conclusion:**

ODD for the treatment of glaucoma were broadly deemed acceptable by patients in this study. Effectiveness and long-lasting effect were the most important factors for a decision towards having an ODD. The majority of patients who preferred an ODD felt severely affected by their disease and were negatively influenced by their glaucoma medication intake.

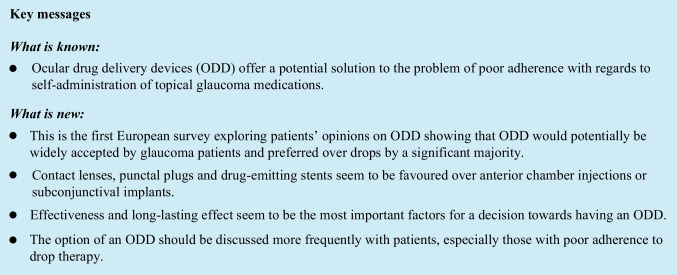

**Supplementary Information:**

The online version contains supplementary material available at 10.1007/s00417-023-06248-1.

## Introduction

The only currently effective treatment for chronic open-angle glaucoma (COAG) consists of lowering intraocular pressure (IOP) with individual target pressures varying for each patient [1–3]. Initial therapy usually consists of topical medication and/or selective laser trabeculoplasty (SLT) [4]. Poor adherence to IOP-lowering topical medication can lead to visual field progression and deterioration of glaucoma in a significant number of cases [5]. The majority of glaucoma patients do not take their medications as prescribed and adherence in glaucoma patients is even lower when compared to other chronic conditions [6, 7]. A recent cost-utility analysis showed that patients non-adherent to glaucoma drops developed unilateral blindness years earlier than those engaged in their treatments[8].

Ocular drug delivery devices (ODD) offer a potential solution to the problem of poor adherence with regard to self-administration of topical glaucoma medications [9]. These devices include contact lenses (CLs) [10, 11], punctal plugs (PPs) [12], subconjunctival implants [13], intracameral implants [14], and drug-emitting stents[15], each delivering ocular anti-glaucomatous medications over varying time frames. Two previous studies from Singapore demonstrated high acceptance for subconjunctival implants as eye-drops replacement, with patients even willing to accept higher costs for such treatments [16, 17]. Our study was performed to evaluate patients’ beliefs towards glaucoma adherence and illness perception and their acceptance towards ODD within a busy German university hospital glaucoma clinic. To our knowledge, this is the first ever survey on ODD involving patients from continental Europe.

## Materials and methods

### Study design

This was a cross-sectional study performed in the glaucoma clinic of a busy tertiary referral hospital in Germany. Ethical approval was obtained by the local ethics committee of the University Hospital Bonn (reference: 185/22). The study protocol conformed to the ethical guidelines of the 2000 Declaration of Helsinki as reflected in a priori approval by the institution’s Human Research Committee. Several pilot questionnaires were conducted between March and May 2022 to adjust and finalize the questions according to feedback received from multiple test runs. Volunteers were recruited in clinic with questionnaires explained and delivered by three trained interviewers who were also available for patient support. Before starting the questionnaire, we handed out a patient information sheet informing patients about the possibility of ODD and explaining their development and purpose. The different ocular drug delivery devices were explained in detail within the questionnaire with images showing an example of each and any remaining questions pertaining to a device were answered by the trained interviewers in particular. The questionnaires were in German, and all patients participated anonymously after giving fully informed consent. Recruitment took place between June and December 2022.

### Inclusion and exclusion criteria

We included patients aged 18 or above with all types of glaucoma who were on at least one topical glaucoma medication for 6 months or more prior to participating. Exclusion criteria included inability to read/understand the questionnaire, refusal to participate, and previous glaucoma surgery.

### Structure of questionnaire

The first section collected socio-economic data and details about topical medication and administration. We included three validated questionnaires that were modified slightly for the study purposes:

*The Beliefs about Medicines Questionnaire (BMQ)* [18] consisting of two parts: BMQ-specific, which investigates the prescribed medication and concerns in that regard and BMQ-general, which looks at attitudes towards medicines in general. We modified both parts in view of patients’ glaucoma medication and utilized both parts for our questionnaire (see Appendix). We simplified the BMQ with answer choices of “I agree” or “I disagree” to facilitate replies as the original version did not work well in our test run.

*The Brief Illness Perception Questionnaire (BIPQ)* assesses the cognitive and emotional representation of illness via a nine-item scale. It has a good test–retest reliability and validity and is useful to conduct a rapid assessment of illness perception [19]. The BIPQ asks patients to circle a number between 0 and 10 that best corresponds to their view on a certain statement. It was translated into German and used without changes (see Appendix).

*The Modified 8-Item Medication Adherence Scale (MMAS-8)* [20] is a validated, structured test to assess self-reported medication adherence, originally developed for arterial hypertension patients. We modified this with a glaucoma medication/disease perspective (see Appendix).

The last section was self-designed and adapted from the above-mentioned Chan study from Singapore[21]. Every ODD was explained briefly with knowledge and acceptance regarding ODD (CLs, PPs, subconjunctival implants, anterior chamber (AC) injections, and drug-emitting stents) assessed. We aimed to investigate factors that could influence patients’ decisions in choosing a certain ODD and compared these five specific types of ODD with each other.

### Statistical analysis

Statistical analysis was performed with SPSS Statistics version 27.0.0 (IBM Corporation, New York). The Pearson’s chi-square test was used to compare the distributions of nominal and ordinal scaled variables. The *t*-test was used for normal distributions and Mann–Whitney *U*-test for non-normal distributions in order to compare two independent groups. Univariate all tests were performed two-sided, and we considered *P* values < 0.05 to be statistically significant.

## Results

Of 206 eligible patients, 152 (73.8%) were willing to participate in the study and 102 (49.5%) successfully completed the questionnaire. Forty-four patients (43.1%) were male. The majority of patients (94, 92.2%) had public, subsidized insurance. Twenty-five patients (24.5%) stated they lived alone at home, and 89 patients (87.3%) self-administered their eye-drops. The mean time that patients needed to get to our eye clinic was 1.13 h. All 102 patients were using IOP-lowering drops, with 46 (45.1%) confirming having had side-effects from them at some point. We asked patients to rank the degree of side-effects on a scale from zero to ten (see Appendix). The mean degree of side-effects was 2.44/10. Side-effects included dry eye symptoms (18.6%), red eye (10.8%), visual deterioration (9.8%), pain (2.0%), and others (7.8%), amongst which headaches. Table 1 describes further patient characteristics.

**Table 1:** Patient characteristics

Variable	Patients *n* = 102, %
Age	
18–40	9, 8.8
41–60	32, 31.4
61–80	51, 50
> 80	10, 9.8
Male	44, 43.1
Time to eye hospital (h)	1.13 (0.723)
Transport medium to eye hospital	
Car	23, 22.5
Public transport	23, 22.5
Someone drives me	46, 45.1
Others	10, 9.8
Subsidized patient	94, 92.2
Ethnicity	
Caucasian	101, 99
Afroamerican	1, 1
Religion	
None	22, 21.6
Christian	78, 76.5
Islam	2, 2.0
Education level	
Finished high school	66
Graduated from university	24
Job	
Professional, executive, and managerial	8, 7.8
Self-employed	8, 7.8
Production, technical, mechanical	10, 9.8
Housewife	2, 2.0
Clerical, administrative	7, 6.9
Retired	41, 40.2
No occupation	3, 2.9
Others	23, 22.5
Alone at home	25, 24.5
Monthly income	
0–2000 €	30, 29.4
2001–5000 €	41, 40.2
5001–9999 €	6, 5.9
10000 € and more	1, 1
none	4, 3.9
Undisclosed	20, 19.6
Administration of drops by themselves	89, 87.3
No. of other medication (mean, SD)	1.93 (1.879)
Side-effects from pressure-lowering eye drops: yes	46, 45.1
Degree of side effects: mean, SD	2.436 (1.44)
Which side effects?	
Dry eye	19, 18.6
Visual deterioration	7, 9.8
Red eye	11, 10.8
Pain	2, 2.0
Others	7, 9.8

In terms of patient beliefs regarding their glaucoma medication (Table 2), the majority felt that their health depended on their eye-drops (79.4%) and that eye-drops “protected them” (90.2%).

**Table 2:** Beliefs in medicine: I agree

Specific — necessity	
My health depends on my eye-drops	81, 79.4%
My life would be impossible without eye-drops	41, 40.2%
My eye-drops protect me	92, 90.2%
Having to take eye-drops worries me	42, 41.2%
Specific — concerns	
I do not understand how my eye-drops work	14, 13.7%
My eye-drops disrupt my life	36, 35.3%
I worry about becoming too dependent on eye-drops	28, 27.5%
General — Harm scale	
Most medicines are addictive	24, 23.5%
Medicine do more harm than good	8, 7.8%
All medicine are harmful	3, 2.9%
General — overuse scale	
Natural remedies are safer than medicine	12, 11.8%
Doctors place too much trust on medicine	31, 30.4%
If doctors had more time, they would prescribe fewer medications	48, 47.1%
General — benefits scale	
Medicine help people to live better lives	89, 87.3%
Benefits outweigh the risks	91, 89.2%

Some patients stated that their life was disrupted by eye-drops (35.3%) and that they were worried about eye-drop intake (41.2%). Patients in our study felt that medicines in general help people live better lives (87.3%) with benefits outweighing their risks (89.2%). Nevertheless, nearly half (47.1%) stated that they believe doctors would prescribe fewer medications if they had more time for their patients.

With regard to the BIPQ (Table 3), patients felt that glaucoma had a moderate impact on their lives (mean value 4.83/10 scale). The majority believed that their glaucoma would continue forever (9.07 mean value). Patients said they were rather concerned about their glaucoma (7.16 mean value) and moderately affected emotionally (4.12 mean value).

**Table 3:** Brief illness perception: mean values (standard deviation)

How much does your glaucoma affect your life?	4.83 (2.678)
How long do you think your glaucoma will continue?	9.07 (2.168)
How much control do you feel you have over your glaucoma?	4.88 (2.822)
How much do you think your treatment can help your glaucoma?	7.45 (2.095)
How much do you experience symptoms from glaucoma?	4.45 (2.971)
How concerned are you about your glaucoma?	7.16 (2.315)
How well do you feel you understand your glaucoma?	7.46 (2.319)
How much does your illness affect you emotionally?	4.12 (3.065)

As to medication adherence (Table 4), the majority stated that they applied their eye-drops regularly, with 63 patients (61.8%) ng so 90–99% of the time and 31 patients (30.4%) 70–89% of the time. The majority reported that they either never (31.4%) or seldomly (50%) forgot their eye-drops whilst fewer patients stated that this was sometimes (9.8%) or always (2.9%) the case. Thirty-seven patients (36.3%) felt stressed by the use of their glaucoma medication.

**Table 4:** Medication adherence

Eyedrop intake	
100%	2, 2.0%
90–99%	63, 61.8%
70–89%	31, 30.4%
50–69%	3, 2.9%
< 50%	3, 2.9%
Modified 8-item medication adherence scale	
Forget eye drops sometimes: yes	40, 39.2%
Not taken eye drops during the last 2 weeks: yes	15, 14.7%
Ever not taken eye drops: yes	8, 8%
Forgotten eye drops when not at home: yes	24, 23.5%
Take eye-drops yesterday: yes	95, 93.1%
Stopped eye-drops because glaucoma felt well-controlled: yes	5, 4.9%
Stressed by glaucoma medication	37, 36.3%
Forgotten drops	
- never	- 32, 31.4%
- seldom	- 51, 50%
- for a short time	- 6, 5.9%
- sometimes	- 10, 9.8%
- all the time	- 3, 2.9%

### Patients’ opinion on ODD

Our survey demonstrated high acceptance levels towards different ODD (Table 5).

**Table 5:** Patients ‘ opinion on ODD

Variable	Patients *n* = 102
Acceptance of ODD	
Contact lenses	
Have you heard of it?	9, 8.7%
How helpful would it be (mean, SD)?	5.77 (3.115)
Would you accept this option?	49, 48.0%
Punctal plugs	
Have you heard of it?	2, 2.0%
How helpful would it be (mean, SD)?	5.81 (3.02)
Would you accept this option?	54, 52.9%
Subconjunctival implants?	
Have you heard of it?	1, 1%
How helpful would it be (mean, SD)?	5.34 (2.90)
Would you accept this option?	37, 36.3%
Anterior chamber implant	
Have you heard of it?	3, 2.9%
How helpful would it be (mean, SD)?	5.30 (2.93)
Would you accept this option?	29, 28.4%
Stent	
Have you heard of it?	23, 22.5%
How helpful would it be (mean, SD)?	5.97 (3.036)
Would you accept this option?	45, 44.1%
Acceptance of ODD	
Keep drops / Want ODD	39, 38.2%; 63, 61.8%
Different decision, if ODD was done in operation room	31, 30.4%
Factors influencing decision: mean value (SD)	
Personal costs	5.79 (3.151)
Costs for hospital	4.35 (2.92)
Effectivity	9.38 (1.29)
Less side effects	8.81 (1.79)
Less control visits	8.44 (2.46)
Reversibility	7.89 (2.47)
Biodegradibility	8.02 (2.21)
Do-it yourself	5.96 (3.02)
Long-lasting effect	9.05 (1.69)
Which would be the most important for you (pick two)?	
Personal costs	4, 3.9%
Costs for hospital	10, 9.8%
Effectivity	79, 77.5%
Less side effects	9, 8.8%
Less control visits	7, 6.9%
Reversibility	1, 1.0%
Biodegradibility	8, 7.8%
Do-it yourself	71, 69.9%
Long effect	1, 1.0%
Which effect would treatment costs have for you if treatment was more expensive than your current treatment?	
Irrelevant	48, 47.1%
Choose another option	*10, 9.8%*
Depends on the costs	44, 43.1% - between 20 and 2000 €
If every treatment was equally effective, which one would you choose?	
Contact lenses	41, 40.2%
Punctal plugs	*23, 22.5%*
Subconjunctival implants	6, 5.9%
Anterior chamber implant	*6, 5.9%*
Stent	26, 25.5%
If efficacy and invasiveness increased with the procedures below?	
Contact lenses	20, 19.6%
Punctal plugs	16, 15.7%
Subconjunctival implants	14, 13.7%
Anterior chamber implant	9, 8.8%
Stent	43, 42.2%
If duration and invasiveness increased with the procedures below?	
Contact lenses	19, 18.6%
Punctal plugs	15, 14.7%
Subconjunctival implants	11, 10.8%
Anterior chamber implant	8, 7.8%
Stent	49, 48.0%

A large share of patients would accept CLs (48.0%), PPs (52.9%), or stents (44.1%), whilst subconjunctival (36.3%) or AC implants (28.4%) were less accepted. Sixty-three patients (61.8%) confirmed that they would rather have an ODD than keep their eye-drops. Of these however, 31 stated that they would reconsider if the ODD was done in the operation room.

The most important factors that influenced preference were effectivity (mean value: 9.38/10) and long-lasting effect (mean value: 9.05/10). Other important factors included less side-effects (8.81/10), less follow-up visits (8.44/10), biodegradability (8.02/10), and reversibility (7.89/10). Factors such as personal costs (5.79/10), costs for the hospital (4.35/10), or a “do-it-yourself” option (5.96/10) were considered less important. When asked to choose the *two most important* factors, the majority of patients chose effectivity (77.5%) and, in contrary to their grading, the “do-it-yourself” option (69.9%). Forty-eight patients (47.1%) stated that ODD costs would be irrelevant, whilst 44 (43.1%) felt their decision would depend on cost. There was no significant correlation to patient income (*p* = 0.218) when comparing those with 0–1999€, 2000–4999€, and 5000€ per month or more. The financial sums these patients were willing to pay themselves ranged from €20 to €2000 (mean €349.49).

If each of the five ODD were equally effective, most patients would want CLs (40.2%) or stents (25.5%). If efficacy + invasiveness or duration + invasiveness increased in accordance with their order in the questionnaire, 42.2% and 48.0% would then choose a stent.

### Comparison of patients who would prefer keeping eye-drops (group-1) versus patients preferring an ODD (group-2)

Table 6 details these results. There were no major differences regarding age (*p* = 0.147), sex (*p* = 0.245), travelling time to hospital (*p* = 0.878), ethnicity (*p* = 0.711), education level (*p* = 0.456), monthly income (*p* = 0.145), or insurance type (*p* = 0.711). More patients in group-2 had experienced eye-drop side-effects (group-1: 33.3% vs. group-2: 52.4%, *p* = 0.108) with the degree also being significantly more severe in this group (group-1: 1.47 vs. group-2: 3.03; *p* = 0.006). As to religious beliefs, a significantly higher number in group-2 did not have any, whilst more group-1 patients were Christian (*p* = 0.020).

**Table 6:** Comparison of patients that would prefer to keep their drops vs. patients that would prefer ODD

Variable	Prefer drops *n* = 39, 38.2%	Prefer ODD *n* = 63, 61.8%	*p*
Age			0.147
18–40	2, 5.2%	7, 11%	
41–60	8, 20.5%	24, 38.1%	
61–80	22, 56.3%	29, 46%	
> 80	7, 18%	3, 4.8%	
Male	14, 31.8%	25, 43.1%	0.245
Side effects: yes	13, 33.3%	33, 52.4%	0.108
*Degree of side effects*	*1.47 (2.273)*	*3.03 (2.97)*	*0.006*
Which side effects?			0.272
Dry eye	7, 18.4%	12, 19.4%	
Visual deterioration	2, 5.2%	5, 7.9%	
Red eye	2, 5.2%	9, 14.3%	
Pain	0	2, 3.2%	
Others	2, 5.3%	5, 8.1%	
Time to Eye Hospital (h)	1.13 (0.751)	1.15 (0.701)	0.878
Subsidized patient	36, 92.3%	58, 92.1%	0.711
Ethnicity			
Caucasian	39, 100%	62, 98.4%	
Afroamerican		1, 1.6%	
Religion			0.020
*None*	*3, 7.7%*	*21, 33.3%*	
*Christian*	*36, 92.3%*	*41, 65.1%*	
*Islam*	*0*	*1, 1.6%*	
Education level			0.456
Finished high school	22, 56.3%	44, 69.8%	
graduated from university	10, 25.7%	14, 22.2%	
Alone at home	9, 23.1%	16, 25.4%	0.697
Monthly income			0.145
0–2000 €	14, 35.9%	16, 25.4%	
2001–5000 €	12, 30.8%	29, 46%	
5001–9999 €	1, 2.6%	5, 7.9%	
10,000 € and more	0	1, 1.6%	
none	1, 2.6%	3, 4.8%	
Undisclosed	11, 28.1%	9, 14.3%	
Administration of drops by themselves	32, 84.2%	57, 90.5%	0.359
No. of other medication (mean, SD)	1.13 (0.72)		0.472
Beliefs in medicine: I agree			
My health depends on my eye-drops	33, 84.6%	48, 76.2%	0.306
My life would be impossible without eye-drops	19, 48.7%	22, 34.9%	0.212
My eye-drops protect me	37, 94.9%	55, 87.3%	0.364
*Having to take eye-drops worries me*	*6, 15.4%*	*36, 57.1%*	< *0.0001*
*I do not understand how my eye-drops work*	*1, 2.6%*	*13, 20.6%*	*0.024*
*My eye-drops disrupt my life*	*3, 7.7%*	*25, 39.7%*	< *0.0001*
*I worry about becoming too dependent on eye-drops*	*3, 7.7%*	*25, 39.7%*	< *0.0001*
Most medicines are addictive	9, 23.1%	15, 23.8%	0.932
Medicine do more harm than good	1, 2.6%	7, 11.1%	0.119
All medicine are harmful	0	3, 4.8%	0.167
Natural remedies are safer than medicine	3, 7.7%	9, 14.3%	0.315
*Doctors place too much trust on medicine*	*6, 15.4%*	*25, 39.7%*	*0.010*
If doctors had more time, they would prescribe fewer medications	13, 33.3%	35, 55.6%	0.710
Medicine help people to live better lives	33, 84.6%	56, 88.9%	0.529
Benefits outweigh the risks	35, 89.7%	56, 88.9%	0.892
Brief Illness Perception: mean values (standard deviation)			
*How much does your glaucoma affect your life?*	*3.9 (2.521)*	*5.41 (2.625)*	*0.005*
How long do you think your glaucoma will continue?	9.56 (1.603)	8.76 (2.414)	0.069
*How much control do you feel you have over your* glaucoma*?*	*5.79 (2.811)*	*4.32 (2.699)*	*0.010*
How much do you think your treatment can help your glaucoma?	7.54 (2.088)	7.40 (2.114)	0.742
How much do you experience symptoms from glaucoma?	3.92 (2.941)	4.78 (2.965)	0.159
How concerned are you about your glaucoma?	6.79 (2.105)	7.38 (2.426)	0.216
How well do you feel you understand your glaucoma?	7.67 (1.991)	7.33 (2.508)	0.460
How much does your illness affect you emotionally?	3.41 (2.926)	4.56 (3.089)	0.066
Medication adherence			
Eyedrop intake			0.169
100%	0	2, 3.2%	
90–99%	28, 71.8%	35, 55.6%	
70–89%	9, 23.1%	22, 34.9%	
50–69%	0	3, 4.8%	
< 50%	2, 5.2%	1, 1.6%	
*Forget eye drops sometimes: yes*	*10, 25.6%*	*30, 47.6%*	*0.027*
*Not taken eye drops during the last 2 weeks: yes*	*1, 2.6%*	*14, 22.2%*	*0.016*
*Ever not taken eye drops: yes*	*0*	*8, 12.7%*	*0.020*
Forgotten eye drops when not at home: yes	6, 15.4%	18, 28.6%	0.213
Take eye-drops yesterday: yes	37, 94.9%	58, 92.1%	0.586
Stopped eye-drops because glaucoma felt well-controlled: yes	0	5, 7.9%	0.139
*Stressed by glaucoma medication*	*8, 20.5%*	*29, 46.0%*	*0.021*
Acceptance towards Ocular drug delivery devices			
*Contact lenses: yes*	*15, 38.5%*	*38, 60.3%*	*0.032*
Punctal plugs: yes	16, 41%	38, 60.3%	0.058
Subconjunctival Implant: yes	12, 30.8%	25, 39.7%	0.363
*Anterior chamber implant: yes*	*5, 12.8%*	*24, 38.1%*	*0.006*
*Stent: yes*	*11, 28.2%*	*34, 54.0%*	*0.023*
Importance of different factors for choice of ODD: mean values (standard deviation)			
Personal costs	5.23 (3.280)	6.14 (3.042)	0.156
Costs for hospital	3.69 (2.87)	4.76 (2.894)	0.072
*Effectivity*	*9.0 (1.792)*	*9.62 (0.771)*	*0.018*
*Less side effects*	*8.23 (0.344)*	*9.17 (0.144)*	*0.009*
Less control visits	6.79 (3.019)	5.65 (1.795)	0.402
Reversibility	7.54 (2.713)	8.11 (2.301)	0.257
Biodegradibility	7.87 (2.226)	8.11 (2.208)	0.451
Do-it yourself	*8.54 (2.037)*	*9.37 (1.274)*	*0.013*
*Long effect*	6.62 (2.672)	5.56 (3.176)	0.085
If every treatment was equally effective, which one would you choose?			0.803
Contact lenses	15, 38.5%	26, 41.3%	
Punctal plugs	10, 25.6%	13, 20.6%	
Subconjunctival implants	3, 7.7%	3, 4.8%	
Anterior chamber implant	3, 7.7%	3, 4.8%	
Stent	8, 20.5%	18, 28.6%	

There were notable differences between groups in terms of patients’ beliefs: Significantly more patients in group-2 were worried by their eye-drop intake (*p* < 0.001), did not understand their eye-drops (*p* = 0.0024), felt disrupted by them (*p* < 0.001), or were worried about becoming too dependent on them (*p* < 0.001). Moreover, patients in group-2 felt more strongly that doctors place too much trust in medicine (*p* = 0.010). Patients in group-2 significantly felt that their lives were more severely affected by their glaucoma (*p* = 0.005) and that they had less control over their disease (*p* = 0.010). Patients in group-2 reported forgetting their drops ‘at least sometimes’ more than those in group-1 (*p* = 0.027). Moreover, more patients in group-2 reported not having taken their eye-drops within the previous two weeks (*p* = 0.016) or ever (*p* = 0.020). A significantly greater share felt stressed by taking their glaucoma medication in group-2 compared to group-1 (*p* = 0.021).

There were significant differences between groups regarding acceptance of different ODD: A greater share showed acceptance towards CLs (group-1: 38.5% vs. group-2: 60.3%; *p* = 0.032), AC implants (group-1: 12.8% vs. group-2: 38.1%, *p* = 0.006), or stents (group-1: 28.2% vs. group-2: 54%, *p* = 0.023). There were no significant differences regarding acceptance of PPs (*p* = 0.363) or subconjunctival implants (*p* = 0.058). Factors graded significantly different were effectivity (mean group-1: 9.0; group-2: 9.62; *p* = 0.018), less side-effects (mean group-1: 8.23; group-2: 9.17; *p* = 0.009), and long-lasting effect (mean group-1: 8.54; group-2: 9.37; *p* = 0.013).

## Discussion

Non-adherence to topical glaucoma medications can lead to disease progression with concomitant irreversible visual field loss. As clinicians, it is essential to keep this problem in mind and to remember that patients may feel disturbed or worried by their medication use. It is easy for us to assume that we know better than our patients and that perhaps other options such as ODD may be deemed ineffective, too invasive, or simply too expensive. The first thing that our survey demonstrates is a clear majority (62%) of patients favoring an ODD over the use of eye drops.

The idea of exploring patients’ perspectives on ODD is not completely new. In 2015, Chan et al. performed a survey in a Singaporean glaucoma population, looking at subconjunctival, intracameral and PP routes as ODD[21]. The majority of interviewed patients accepted at least one of the three alternatives; PPs being the preferred route in this cohort whilst “effectiveness” was the most important factor determining the actual choice. Due to the further development and broader selection of ODD, we included five different choices: CLs, PPs, subconjunctival implants, AC injections and drug-emitting stents, based on the literature and studies performed to evaluate these different ODD types. We included a brief description of these various options, including specific availability, evidence, and potential side-effects, in order to enable informed patient responses [22–28]. Compared to Chan et al., we incorporated similar (albeit not exactly the same) question strategies. However, a main difference is that patients in Singapore “co-pay” for their health care system, whereby costs for citizens with lower socioeconomic standing are subsidized. In Germany, a compulsory health insurance exists, with patients usually subsidized without additional costs, with an option to purchase additional private insurance if desired.

The BMQ outcomes in our study showed that the majority of patients felt that their eye-drops protected them (90.2%) and that their health depended on them (79.4%), depicting a positive perception of glaucoma medications in most patients. On answering the BIPQ, patients highly rated the questions about whether their treatment could help their condition and if they understood their disease, underlining their understanding of glaucoma and the fact that treatment reduces progression. Chan et al. had similar findings stating that patients felt their medication delivered a positive benefit. This understanding is of course essential for patients with a chronic disease such as glaucoma and can influence their adherence behaviors. On the other hand, a considerable proportion of patients interviewed were worried or felt disrupted by eye-drop intake (41.2%, 35.3%), rating their concern about glaucoma at 7.15. This shows that although most patients know that eye-drops are necessary to protect their health, many of them worry about drop intake, whilst also being concerned about their disease. Nearly half of responders believed that doctors would prescribe fewer medications if they had more time, denoting the potential perception that drops are prescribed to appease patients and facilitate patient turnover in busy clinics. As clinicians we should therefore invest in time explaining why medication is prescribed and in ways of highlighting the importance of adherence to self-administered therapy. We specifically included the MMAS-8 questionnaire to obtain real-world data on the latter with a considerable number stating that they sometimes forget their eyedrops (39.2%), forget them when not at home (23.5%), or feel stressed by their medication (36.3%). This confirms the long-known fact that glaucoma patients’ eye-drop adherence is sub-optimal, particularly as effectiveness relies on regular administration [29]. As a consequence, clinicians may often get a false idea of actual IOP values or medication effectiveness, either because of seemingly controlled IOP (in those who only medicate on the day of an appointment) or high IOP (for those missing drop administration).

ODD will almost certainly evolve, becoming more available and popular over time [30]. However, most of our patients had not heard of any ODD alternatives to eye-drops (see Table 5) despite the survey being conducted within a large university hospital, known to be an active investigator and implementer of new devices. People who only have access to local ambulatory health care might be even less aware of such alternatives. Our patients’ paucity of knowledge on ODD may also reflect limited awareness amongst ophthalmologists. Many clinicians would probably commence or maintain topical IOP-lowering therapy despite known side-effects, possibly as they are still considered the least invasive option. The advent of SLT, including trials promoting it as first line therapy [31], has not really dented the large proportion of glaucoma patients still treated with eye-drops. Whereas many clinicians may not have heard of the various ODD available, others might just consider them more invasive in comparison to drops or SLT. It is thus our duty as glaucoma specialists to inform both patients and colleagues on any available or forthcoming ODD modalities so that they can weigh the benefits and risks for each individual patient.

Nearly half of our patients felt that costs would be irrelevant (47.1%) in the context of ODD preference. However, we must consider that many glaucoma patients may actually not afford the additional expense. A cross-sectional study in the USA showed more cost-related non-adherence amongst glaucoma patients compared to participants without glaucoma, underlining the importance of cost considerations and their impact on therapy adherence [32]. Our surveyed patients stated that they were willing to pay from between €20 and €20,000 extra for an ODD, evidencing a huge variance between additional funding patients may have beyond health insurance cover. The majority of our interviewed patients (92.2%) had subsidized insurance with most on a monthly income of €0–€2000 (29.4%) and €2001–€5000 (40.2%) with additional treatment costs implying they would have to draw on significant savings. Clearly there is a discrepancy between people’s wishes and what they could really afford. The mainstay for ODD development has been the drive to remove patient adherence issues. Ironically, the inhibiting cost may become a determinant factor against this becoming an effective strategy.

Our comparison between patients preferring eye-drops versus those favoring an ODD showed that the latter suffered significantly more from drop side-effects (*p* = 0.006). These patients would therefore be the main beneficiaries of an ODD. Patients who reported worries about their drop intake stated that they did not understand how the drops worked, felt disrupted by drops and/or were worried about becoming too dependent on them. These patients preferred ODD more often in our survey. Patients who preferred ODD also had a significantly more negative perception of their disease: they felt more severely affected by their glaucoma and that they had less control of their condition. Perhaps the BMQ and BIPQ may be a good modality to understand which patients struggle more with glaucoma drops and might identify preferable candidates for an ODD.

When considering an ODD intervention, it is important to evaluate which ODD would best suit an individual patient. If each treatment was equally effective, the majority would prefer CLs (40.2%) followed by stents (25.5%) and PPs (22.5%). A possible explanation for this selection would be that CLs are commonly used or known by many people in the context of refractive error correction. Furthermore, CLs can be inserted independently and outside a clinic setting. PPs also showed a high acceptance overall, although this was less popular compared to the CLs option. This might be explained by the fact that PPs need to be administered by the eye specialist. Moreover, patients would not be too familiar with this way of administration compared to CLs. In comparison to the three other ODD options in our questionnaire, PPs and CLs may have been preferred as they do not involve needles, sharp objects or higher degrees of invasiveness. Although stents were the second preferred option, 30.4% of patients stated they would reconsider their decision if the intervention was performed in the operating room. When comparing the three “more invasive” procedures, stents seemed to be more widely accepted than subconjunctival or AC implants. This could be attributed to the fact that far more patients (22.5%) had already heard of a “stent” option whilst less than 10% had heard of the other alternatives, and therefore more comfortable with a more familiar type of procedure. When asked whether preference would shift to a more invasive option should this be more effective or longer lasting, our patients replied affirmatively, with the majority preferring a stent in this scenario.

Our study has various limitations. Firstly, it is a single-center study with limits to extrapolating the results to a wider regional, national or global population. Furthermore, 102 patients represent a relatively small number of participants, so that a certain selection bias may be present. The latter is also the case in terms of which patients might be more inclined to accept the invitation of participating in such a survey. An international, multicenter study, which we currently have underway, reflecting different patient characteristics and larger numbers, would be more able to discern differences between various centers and countries. We also did not gather visual field data on the patients surveyed and therefore could not classify them into actual severity types. However, we did not recruit patients with known advanced disease or those having undergone previous glaucoma surgery. Another limitation of such a survey is the limited knowledge related to ODD in this case. We tried addressing this by including different descriptions of the various ODD to offer patients a basic foundation on which to base answers. Although these descriptions were completely objective, a certain bias may always be present, and the images and wording used may in themselves introduce this.

Moreover, the majority of our patients had not heard of ODD prior to participating in the questionnaire. This can lead to a certain selection bias depending on how ODD were explained to the patients. In order to counteract this problem, we handed out patient information sheets before starting the questionnaire which introduced ODD in general to our patients to give them information. The different ODD were then explained in detail within the questionnaire with images showing the different ODD and our trained interviewers were available for questions. We attempted to keep descriptions, images (see Appendix) and explanations as objective and realistic as possible in order to not affect patients’ decisions and opinions towards certain ODD. Our trained interviewers were told to not influence patients and state an opinion of their own. However, a remaining bias or influence could have still been present.

In conclusion, this survey strongly confirms that many patients are non-adherent to topical glaucoma medications and feel worried and negatively influenced by their drop treatment and disease. The study also shows that there is a potentially high acceptance towards the possibility of ODD as alternatives to drop treatment, with PPs, CLs, and drug-emitting stents being the most popular in our patient cohort. Our patients reported having little knowledge on ODD, highlighting the need for better information on these options for both patients and clinicians. Patients with sub-optimal adherence to glaucoma topical medications may benefit from the option of an ODD, with the choice of which being made individually for every patient after thorough discussion and consideration of the risks and benefits of each modality.

### Supplementary Information

Below is the link to the electronic supplementary material.

Supplementary file1 (PDF 5327 KB)

## Data Availability

All datasets generated during and/or analyzed during the current study are available from the corresponding author on reasonable request.
