# Supplementary material for: Ocular drug delivery systems: glaucoma patient perceptions from a German university hospital eye clinic
Source: Graefes Arch Clin Exp Ophthalmol. 2023 Sep 30;262(2):545–56. doi: 10.1007/s00417-023-06248-1 (PMC10844344; doi:10.1007/s00417-023-06248-1)
Supplement: Supplementary file 1 — Supplementary file1 (PDF 5327 KB) [file 417_2023_6248_MOESM1_ESM.pdf]

Patients' Perspectives on Alternative Drug Delivery Systems for Glaucoma Drop Medications

Name of Ref Clinician: Dr. ________________
Date of Informed Consent: ____/____/____
Investigator Code: __________

COUNTRY

Part 1: General patient information concerning glaucoma

1. Do you take glaucoma eye drops?

   ☐ 1 Yes ☐ o No

2. Do you experience any side effects from your glaucoma eye drops?

   ☐ 1 Yes ☐ o No

3. If yes, which ones?

   ____

4. How strong are your side effects on a range from 0 (barely any side effects) to 10 (massive side effects) ?

5. How long does it take you to reach our glaucoma clinic?

   ________ hours

6. How do you get to our clinic?

☐ 1 by car ☐ 2 by public transport
☐ 3 someone drives me ☐ 4 etc
☐ 5 Other, please specify: ___________________________

Part 2: Demographics

1. Are you a subsidized or a private patient?

☐$_1$Subsidized ☐$_2$ Private

2. What is your age?

☐$_1$ 20-30 ☐$_2$ 31-40

☐$_3$ 41-50 ☐$_4$ 51-60

☐$_5$ 61-70 ☐$_6$ 71-80

☐$_7$ 81-90 ☐$_8$ >91

3. What is your ethnicity?

☐$_1$ White (European, American) ☐$_2$ Asian

☐$_3$ Black ☐$_4$ Hispanic

☐$_5$I don't want to give an answer.

☐$_6$ Other, please specify: _______________

Country of origin: ___________________

4. Which religion do you belong to?

☐$_1$ none ☐$_2$ Christian

☐$_3$ Islam ☐$_4$ Judaism

☐$_5$ Buddhism ☐$_6$ Hinduism

☐$_7$ Other, please specify: ___________________________

5. What is your highest level of education?

☐$_1$ No formal education ☐ $_2$ Primary

☐$_3$ Secondary ☐ $_4$ A level

☐$_5$ Polytechnic / Diploma ☐ $_6$ Graduate

☐$_7$ Post Graduate

6. What is your occupation?

☐ 1 Professional, Executive & Managerial
☐ 2 Self-employed
☐ 3 Production, Technical, Mechanical
☐ 4 Housewife
☐ 5 Clerical, Administrative
☐ 6 Retired
☐7 No occupation
☐8 Others, Please specify:

7. What is your monthly income (net income)?

☐1 less than 1,000 €
☐2 1,000 € to less than 2,000 €
☐3 2,000 € to less than 5,000 €
☐4 5,000 € to less than 10,000 €
☐5 10,000 € or more
☐6 I do not know.
☐7 I don't want to give an answer.
☐8 No income..

8. Do you live alone?

☐1 Yes ☐ o No

9. Do you administer your glaucoma eye-drops for yourself?

☐1 Yes ☐ 2 No. ☐3 Sometimes

10. If no or sometimes, who helps you drop your glaucoma eye-drops? (Tick all that applicable)

(a) Husband/Wife/Partner ☐ 1 Yes ☐ o No
(a) Son/Daughter ☐1 Yes ☐ o No
(b) Other relatives/friends/neighbours ☐1 Yes ☐o No
(c) Others, please specify: ______ ☐1 Yes ☐ o No

11. How many oral medications are you on?

☐0

☐1

☐2

☐3

☐4

☐More than 4

8. Are you taking any other medications?

☐$_0$ Sprays (number)

☐$_1$ Injections (number)

☐$_2$ Others, please specify: _______

<u>How I feel about my medicine</u>

1. My health depends on my eye-drops.

☐$_1$ I agree ☐$_2$ I disagree

2. My life would be impossible without my eye-drops.

☐$_1$ I agree ☐$_2$ I disagree

3. My eye-drops protect me from becoming worse.

☐$_1$ I agree ☐$_2$ I disagree

4. Having to take eye-drops worries me

☐$_1$ I agree ☐$_2$ I disagree

5. I do not understand how my eye drops work.

☐$_1$I agree ☐$_2$I disagree

6. My eye-drops disrupt my life

☐$_1$I agree ☐$_2$I disagree

7. I sometimes worry about becoming too dependent on my eye-drops

☐$_1$I agree ☐$_2$I disagree

8. Most medicines are addictive

☐$_1$I agree ☐$_2$I disagree

9. Medicine do more harm than good

☐$_1$I agree ☐$_2$I disagree

10. Most medicines are addictive

☐$_1$I agree ☐$_2$I disagree

11. Natural remedies are safer than medicine

☐$_1$I agree ☐$_2$I disagree

12. Doctors place too much trust on medicine

☐$_1$I agree ☐$_2$I disagree

13. If doctors had more time with patients they would prescribe fewer medicines

☐$_1$I agree ☐$_2$I disagree

14.Medicine help many people to live **better**

☐$_1$I agree ☐$_2$I disagree

15. Medicine help many people to live **longer**

☐$_1$I agree ☐$_2$I disagree

16. In most cases the benefits of medicine outweigh the risks

☐1 I agree ☐2 I disagree

The Brief Illness Perception Questionnaire regarding your glaucoma disease

The Brief Illness Perception Questionnaire

For the following questions, please circle the number that best corresponds to your views:

How much does your illness affect your life?

0	1	2	3	4	5	6	7	8	9	10
no affect at all										severely affects my life

How long do you think your illness will continue?

0	1	2	3	4	5	6	7	8	9	10
a very short time										forever

How much control do you feel you have over your illness?

0	1	2	3	4	5	6	7	8	9	10
absolutely no control										extreme amount of control

How much do you think your treatment can help your illness?

0	1	2	3	4	5	6	7	8	9	10
not at all										extremely helpful

How much do you experience symptoms from your illness?

0	1	2	3	4	5	6	7	8	9	10
no symptoms at all										many severe symptoms

How concerned are you about your illness?

0	1	2	3	4	5	6	7	8	9	10
not at all concerned										extremely concerned

How well do you feel you understand your illness?

0	1	2	3	4	5	6	7	8	9	10
don't understand at all										understand very clearly

How much does your illness affect you emotionally? (e.g. does it make you angry, scared, upset or depressed?

0	1	2	3	4	5	6	7	8	9	10
not at all affected emotionally										extremely affected emotionally

Please list in rank-order the three most important factors that you believe caused <u>your illness</u>. *The most important causes for me:-*

1. ____________________________________

2. ____________________________________

3. ____________________________________

Patient Adherence Questionnaire

How frequently do you take your eyedrops?	☐$_0$ 100%<br>☐$_1$ 90 - 99 %<br>☐$_1$ 51 - 89 %<br>☐$_1$ 0 - 50 %

Taking the prescribed glaucoma medication every day can be very demanding. It is very common to forget taking eye drops from time to time. The following questions are supposed to help to give us an insight on how often patients forget to take their prescribed medication. Please answer them truthfully. Your answers will remain anonymous and your doctor will not get access to your answers.

1. Do you sometimes forget to take your glaucoma eye drops?	☐$_1$Yes<br>☐$_0$ No
2. Over the past two weeks, were there any days when you did not take your glaucoma eye drops?	☐$_1$Yes<br>☐$_0$ No
3. Have you ever cut back or stopped taking your glaucoma eye drops without telling your doctor because you felt worse when you took it?	☐$_1$Yes<br>☐$_0$ No
4. When you travel or leave home, do you sometimes forget to bring along your glaucoma eye drops?	☐$_1$Yes<br>☐$_0$ No
5. Did you take your glaucoma eye drops yesterday?	☐$_1$Yes<br>☐$_0$No
6. When you feel like your glaucoma is under control, do you sometimes stop taking your eye drops?	☐$_1$Yes<br>☐$_0$ No

7. Taking medication everyday is a real inconvenience for some people. Do you ever feel hassled about sticking to your glaucoma eye drops?	☐$_1$Yes<br>☐$_o$ No
8. How often do you have difficulty remembering to take all your glaucoma eye drops?	☐$_0$Never<br>☐$_1$ Very rarely<br>☐$_2$ A little of the time<br>☐$_3$ Sometimes<br>☐$_4$ All the time

Modified 8 item Medication Adherence Scale – Morisky et al., 2008

Part 6: Ocular Drug Delivery (ODD) Questionnaire

The treatment of glaucoma can be very challenging due to the many side effects, especially of eye drops, that may occur and reduce the patient’s quality of life. A treatment option using invasive surgery can cause complications and therefore involves a certain risk.
Glaucoma treatment offers some promising minimally invasive approaches that could improve the treatment outcome and patients’ quality of life. This involves finding alternative ways of applying the medications as an alternative to drops but without major glaucoma surgery being performed. Please imagine you could choose between the following options:

1) Contact lens

A contact lens similar to what people use to replace spectacles can be used to release glaucoma medication. A contact lens is placed onto the surface of the cornea (the clear window on the front of the eye) so it does not involve entering the eye in any way. Certain types of contact lenses can be inserted once, either by yourself or your eye specialist, and remain for weeks/months on the surface of the eye whilst releasing glaucoma medications slowly. The contact lens would have to be replaced at intervals (months) to replenish the medication supply - this can also be done by yourself, carer/partner or eye specialist. This option does not require a visit to the operating room and can theoretically be self-administered at home.

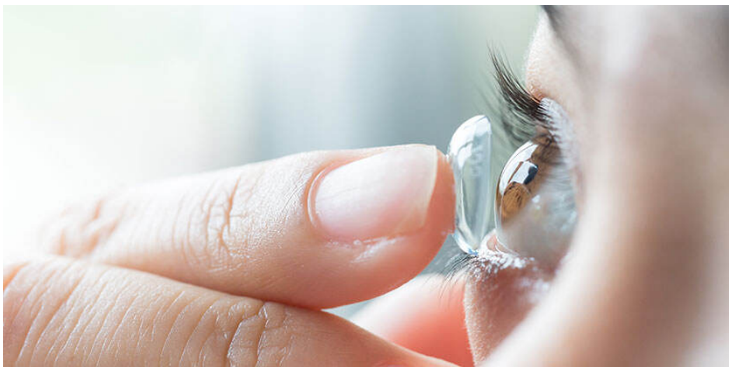

Source: https://www.aao.org/eye-health/diseases/contact_lenses (last accessed 23/04/22)

Have you ever heard of this treatment modality?

☐ $_1$ Yes

☐ $_0$ No

How helpful do you feel this method of treatment would be for the treatment of your glaucoma?

1 2 3 4 5 6 7 8 9 10

Not at all **very helpful**

Would you consider undergoing this type of treatment?

☐ $_1$ Yes

☐ $_0$ No

2) Punctum Plugs

Punctal plugs are tiny devices that are placed in the eye's tear ducts (called puncta). Puncta are the tiny openings that drain tears from your eyes. About the size of a grain of rice, the plug is usually used to stop fluid draining from the eye to treat dry eye conditions but the plug can also be used to slowly release glaucoma medications. Plugs trialled for glaucoma are made of a long-lasting medical plastic (such as silicone or acrylic) and are designed to stay in the eye for many months. They can be removed and replaced by your ophthalmologist if needed. Another type of semi-permanent punctal plug is placed in a deeper part of the tear duct called the canaliculus. These plugs cannot be seen at all in the eye. This option does not require a visit to the operating room but would need an eye specialist to insert/remove and/or ascertain position.

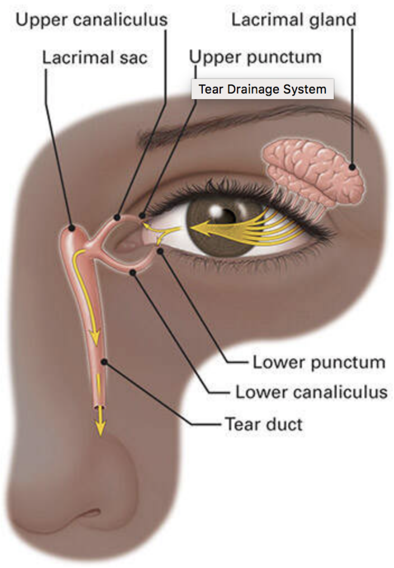

Source: https://www.aao.org/eye-health/diseases/punctal-plugs (last accessed 23/04/22)

Have you ever heard of this treatment modality?

☐ 1 Yes

☐ 0 No

How helpful do you feel this method of treatment would be for the treatment of your glaucoma?

1 2 3 4 5 6 7 8 9 10

Not at all **very helpful**

Would you consider undergoing this type of treatment?

☐ 1 Yes

☐ 0 No

3) Subconjunctival implants

A subconjunctival implant can either be placed under the eyelids or injected beneath the conjunctiva (the transparent cling film layer which covers the sclera (the white of the eye). These implants slowly release glaucoma medications and are covered either by the eyelid or conjunctiva itself, making them less exposed to the external environment when compared to a contact lens or punctal plug.This option does not require a visit to the operating room but would need an eye specialist to insert/remove and/or ascertain position. The injection would involve a very small needle but this is only inserted under the superficial surface of the eye (conjunctiva) and *not into the eye*.

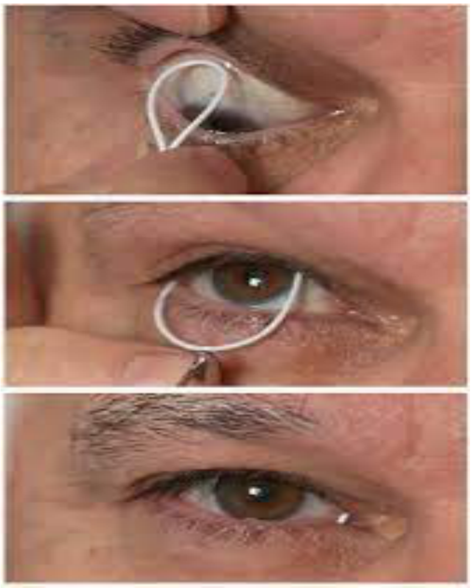

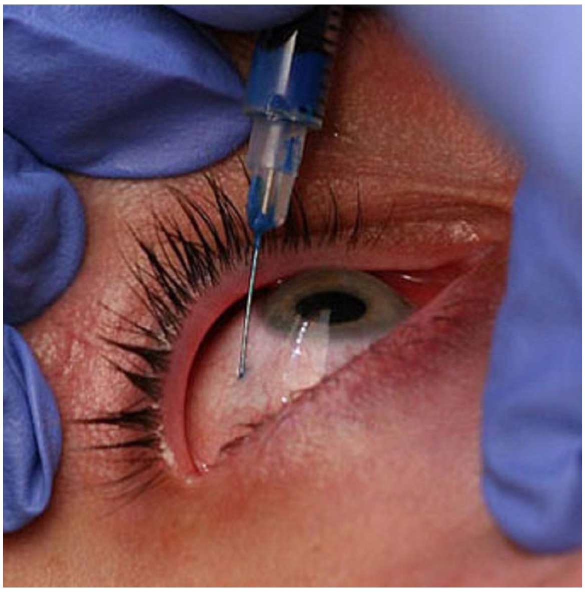

Have you ever heard of this treatment modality?

☐$_1$Yes

☐$_0$No

How helpful do you feel this method of treatment would be for the treatment of your glaucoma?

1 2 3 4 5 6 7 8 9 10

Not at all **very helpful**

Would you consider undergoing this type of treatment?

☐$_1$ Yes

☐$_0$No

4) Anterior Chamber (Intracameral) Administration

Slow release glaucoma medications can be administered into the anterior chamber of the eye (the space between the cornea at the front and iris at the back) via a very small biodegradable, preservative-free implant. Once placed, the drug is slowly released for about 3 to 4 months and the implant slowly dissolves in about 12 to 15 months. The added advantage of this route of administration is that side-effects related to the surface of the eye and skin are significantly reduced compared to drops and even other surface administrations such as mentioned above. On the other hand, the implant is placed into the eye itself, meaning it is slightly more invasive, and it needs to be inserted by an eye specialist in a clean clinic room environment or the operating theatre. Further implantations may/will be needed as the original one loses effect.

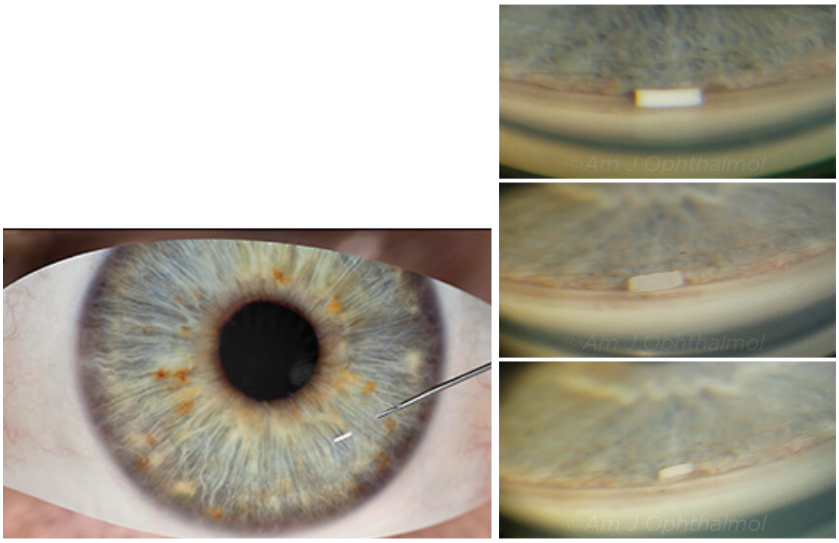

Source: AAO.org

Have you ever heard of this treatment modality?

☐$_1$Yes

☐$_0$ No

How helpful do you feel this method of treatment would be for the treatment of your glaucoma?

1 2 3 4 5 6 7 8 9 10

Not at all **very helpful**

Would you consider undergoing this type of treatment?

☐$_1$ Yes

☐$_0$ No

5) Trabecular Meshwork Microstents

Using a minimally invasive approach an implant/stent can be placed into the anterior chamber of the eye (the space between the cornea at the front and iris at the back) which reduces the intraocular pressure through a mechanical bypass process. These stents have been around for quite a few years now and have been shown to be very effective in reducing the number of glaucoma drops needed by patients. In many cases the stent insertion is combined with cataract surgery (which can also has a pressure lowering effect) but stents can also be used in eyes which have already had cataract surgery or even in eyes with the natural lens still in place (i.e. *not* combined with cataract surgery). Stents are now also being developed with the ability to slowly release glaucoma medications into the anterior chamber of the eye. Although still minimally invasive, stent insertion is more invasive than the above-mentioned options (as it is placed into the eye itself and not onto the surface or superficial layers). It also needs to be performed by an eye surgeon in the operating theatre and may need to be replaced or more stents added later as the effect of the original stent wears off.

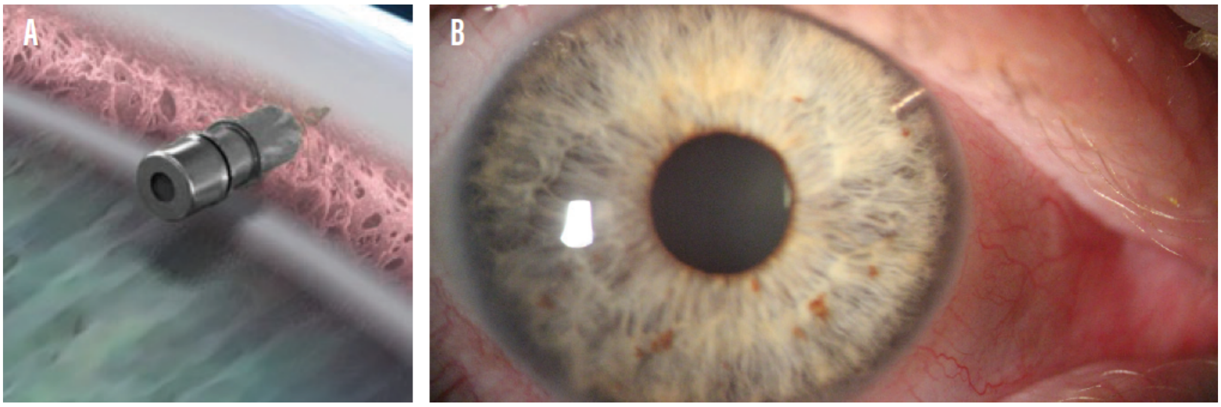

Source:https://glaucomatoday.com/articles/2020-nov-dec/idose-an-intraocular-drug-eluting-delivery-device-for-glaucoma

<u>Have you ever heard of this treatment modality?</u>

☐$_1$Yes

☐$_0$No

<u>How helpful do you feel this method of treatment would be for the treatment of your glaucoma?</u>

1 2 3 4 5 6 7 8 9 10

Not at all **very helpful**

<u>Would you consider undergoing this type of treatment?</u>

☐$_1$ Yes

☐$_0$No

If you had the choice to choose between these different therapy options, would you rather

A: Keep your eye drops
B: Use one or more of the aforementioned therapy options.

Would it make a difference to the answer before if the procedure had to be performed in the operating room?
A: Yes
B: No

Please consider that there are these alternative treatment methods for your glaucoma that could replace the current prescribed eye drops. How important are the following aspects of a treatment modality in your opinion?

A. **HOW IMPORTANT IS EACH OF THE FOLLOWING TO YOU?**

1 2 3 4 5 6 7 8 9 10
Not at all **very important**

Factors	Priority
**Monetary cost** to me	1 2 3 4 5 6 7 8 9 10<br>**Not at all** **very important**
**Monetary cost** to hospital / insurance	1 2 3 4 5 6 7 8 9 10<br>**Not at all** **very important**
**Effectiveness**	1 2 3 4 5 6 7 8 9 10<br>**Not at all** **very important**
**Reduction in side effects**	1 2 3 4 5 6 7 8 9 10<br>**Not at all** **very important**
**Less frequent follow-ups**	1 2 3 4 5 6 7 8 9 10<br>**Not at all** **very important**
**Reversibility of Treatment ***	1 2 3 4 5 6 7 8 9 10<br>**Not at all** **very important**
**Biodegradability ****	1 2 3 4 5 6 7 8 9 10<br>**Not at all** **very important**
**Ability to apply it on your own**	1 2 3 4 5 6 7 8 9 10<br>**Not at all** **very important**
**Long duration**	1 2 3 4 5 6 7 8 9 10<br>**Not at all** **very important**

*** Possibility to take it out without additional risk**
**** It will disappear from your system eventually**

<u>Which of these are the 2 most important points for you? Please tick the two most important ones.</u>

☐ ***Cost***

☐ ***Effectiveness***

☐ ***Reduction in side effects***

☐ ***Less frequent follow-ups***

☐ ***Reversibilty of Treatment***

☐ ***Biodegradability***

☐ ***Ability to apply it on your own***

☐ ***Long duration***

What impact would the cost of the treatment TO YOU have on your decision if the treatment was more expensive than your actual treatment?
A: I don’t care how much it costs.
B: I would not choose this option, but rather go for another treatment.
C: It depends on the difference in cost.

If you ticked C: How much would you be willing to pay:

Have a second to think and ask our interviewer about any doubts

IN THE CASE ALL OF THESE are equally effective, which one would you choose? (only tick 1)

☐1 Contact Lens

☐2 Punctal Plugs

☐3 Subconjunctival

☐4 Intracameral

☐5 Stent

IN THE CASE WHERE EFFICACY AND INVASIVENESS BOTH INCREASED FROM CONTACT LENS THROUGH TO STENTS (I.E. CL LEAST INVASIVE/EFFECTIVE, STENT MOST INVASIVE/EFFECTIVE, which one would you choose? (only tick 1)

☐1 Contact Lens

☐2 Punctal Plugs

☐3 Subconjunctival

☐4 Intracameral

☐5 Stent

IN THE CASE WHERE DURATION AND INVASIVENESS BOTH INCREASED FROM CONTACT LENS THROUGH TO STENTS (I.E. CL LEAST INVASIVE/SHORTEST DURATION/MORE FOLLOW-UPS, STENT MOST INVASIVE/LONGEST DURATION/LESS FOLLOW-UPSwhich one would you choose? (only tick 1)

☐1 Contact Lens

☐2 Punctal Plugs

☐3 Subconjunctival

☐4 Intracameral

☐5 Stent
